# Tyrosine Kinase Inhibitors Stimulate HLA Class I Expression by Augmenting the IFNγ/STAT1 Signaling in Hepatocellular Carcinoma Cells

**DOI:** 10.3389/fonc.2021.707473

**Published:** 2021-08-11

**Authors:** Aya Takahashi, Atsushi Umemura, Kota Yano, Shinya Okishio, Seita Kataoka, Keiichiro Okuda, Yuya Seko, Kanji Yamaguchi, Michihisa Moriguchi, Takeshi Okanoue, Yoshito Itoh

**Affiliations:** ^1^Molecular Gastroenterology and Hepatology, Graduate School of Medical Science, Kyoto Prefectural University of Medicine, Kyoto, Japan; ^2^Department of Gastroenterology and Hepatology, Saiseikai Suita Hospital, Osaka, Japan

**Keywords:** human lymphocyte antigen class I, tyrosine kinase inhibitor, regorafenib, signal transducers and activators of transcription 1, mitogen-activated protein kinase, hepatocellular carcinoma

## Abstract

Combination treatment with tyrosine kinase inhibitors (TKIs) and immunotherapies has shown efficacy in the treatment of multiple cancers, but the immunomodulatory effect of TKIs on the tumor cell phenotype remains unknown in hepatocellular carcinoma (HCC). Given that human lymphocyte antigen class I (HLA-I) is essential for tumor antigen presentation and subsequent antitumor immunity, we examined the effects of regorafenib, as well as other TKIs (sorafenib, lenvatinib and cabozantinib) on HLA-I expression in HCC cell lines. Regorafenib increased cell surface HLA-I and β2-microglobulin protein expression in the presence of interferon γ (IFNγ). The expressions of various genes associated with the HLA-I antigen processing pathway and its transcriptional regulators were also upregulated by regorafenib. Furthermore, we found that regorafenib had an activating effect on signal transducers and activators of transcription 1 (STAT1), and that regorafenib-induced HLA-I expression was dependent on the augmented IFNγ/STAT1 signaling pathway. Trametinib, an inhibitor of the extracellular signal-regulated kinase (ERK) kinase MEK, also activated IFNγ/STAT1 signaling and increased HLA-I expression, whereas the phosphatidylinositol 3-kinase (PI3K) inhibitor buparlisib did not. Given that regorafenib directly inhibits Raf/MEK/ERK signaling, the downregulation of the MEK/ERK pathway appears to be one of the mechanisms by which regorafenib promotes STAT1 activation. Sorafenib, lenvatinib, and cabozantinib also showed the same effects as regorafenib, while regorafenib had most potent effects on HLA-I expression, possibly dependent on its stronger inhibitory activity against the MEK/ERK pathway. These results support the clinical combination of TKIs with immunotherapy for the treatment of HCC.

## Introduction

Hepatocellular carcinoma (HCC) is one of the most common malignant solid tumors and the fourth most frequent cause of cancer-related mortality worldwide (1). HCC is a malignant disease that develops predominantly in patients with underlying chronic liver disease and cirrhosis, thus providing a strong rationale for immune therapy. In recent years, several immune checkpoint inhibitors (ICIs) targeting the programmed death receptor-1 (PD-1) and programmed death ****ligand 1 (PD-L1) pathway have been tested for the treatment of HCC (2). Preliminary results from early-phase clinical trials in HCC indicated a superior treatment response when anti-PD-1 or anti-PD-L1 agents were combined with tyrosine kinase inhibitors (TKIs), including sorafenib, lenvatinib, regorafenib, and cabozantinib (3, 4). The high efficacy of the combination of ICIs and TKIs is not only due to their additive effects on tumor growth, but also to immunomodulatory effects of TKIs. *In vivo* and *in vitro* studies on several types of cancers including HCC demonstrated that TKIs enhanced antitumor immunity by increasing T cell infiltration and function, and regulating immunosuppressive cells such as tumor-associated macrophages, regulatory T cells, and myeloid-derived suppressor cells (5–9). While these effects are linked to modulation of the tumor microenvironment due to an inhibitory effect on vascular endothelial growth factor receptor (VEGFR), the immunomodulatory effect of TKIs on tumor cell phenotypes has not been investigated in HCC.

Human lymphocyte antigen class I (HLA-I) is required for tumor antigen presentation and subsequent cell killing by cytotoxic T lymphocytes (CTLs) and, thus, plays an extremely important role in antitumor immunity (10). Interferon γ (IFNγ) produced by tumor-infiltrating T cells induces signal transducers and activators of transcription 1 (STAT1) activation in tumor cells, promoting HLA-I expression. Therefore, dysfunction of the HLA-I antigen processing pathway (HLA-I APP) or the IFNγ response pathway in tumor cells has been identified as a frequent cause of both primary and acquired resistance to ICIs, often correlating with worse prognosis (11–13). In HCC, it has been reported that the expression of HLA-I is downregulated in 40% to 50% of cancer cells (14–16). Early-stage HCC has sufficient levels of cell surface HLA-I expression, whereas the expression of HLA-I is significantly reduced with increased progression of tumor stage and histological grading of tumor differentiation in HCC tissue (17, 18). Although the mechanism of HLA-I downregulation in HCC is not well understood, treatment with IFNs induces recovery of HLA-I expression in HCC cell lines (19, 20), suggesting that this defect is reversible. Given that CTL recognition of HCC cells is dramatically improved after increasing cell surface HLA-I expression (18, 19), promoting HLA-I expression might be a viable therapeutic approach to enhance the effects of ICIs in HCC.

Sorafenib, lenvatinib, regorafenib, and cabozantinib are broad TKIs that have approved by the FDA for the treatment of advanced HCC. Among them, regorafenib and cabozantinib have been reported to have the ability to increase HLA-I expression in melanoma and colon cancer cell lines, respectively, rendering the cells more sensitive to CTL-mediated killing (5, 8). In addition, various oncogenic pathways have been reported to affect expression of HLA-I in cancer, including mitogen-activated protein kinase (MAPK) and epidermal growth factor receptor (EGFR) (21–23). MAPK pathway activation has been shown to negatively influence HLA-I expression *via* decreased STAT1 expression (22, 24). Hence, downregulation of MAPK signaling by anti-EGFR antibodies, or inhibitors of EGFR, MEK, and BRAF enhances the expression of HLA-I in several types of tumors, such as lung cancer, melanoma, and head and neck squamous cell cancer (21, 22, 25, 26).

This study sought to investigate whether TKIs may also promote the induction of HLA-I molecules in HCC cells. We focused on regorafenib as a model to study the immunomodulatory effect of TKIs because it directly targets not only VEGFR, Tie2, and platelet-derived growth factor receptor (PDGFR), but also BRAF and subsequent MAPK signaling. We found that regorafenib, as well as other TKIs, induces elevated HLA-I expression in HCC cells through enhancement of STAT1 activation by IFNγ, and that downregulation of MAPK signaling might be a mechanism by which TKIs promote HLA-I induction.

## Materials And Methods

### Human HCC Cell Lines and Reagents

The HCC cell lines SNU398, SNU387, Huh7, HepG2, and PLC/PRF/5 were obtained from ATCC. Cells were grown in Roswell Park Memorial Institute (RPMI) medium or Dulbecco’s Modified Eagle’s Medium (DMEM); supplemented with 10% fetal bovine serum (FBS), penicillin, and streptomycin; and maintained at 37°C with a 5% CO2 atmosphere. Regorafenib (#CS-1205) and sorafenib (#CS-0164) were purchased from ChemScene. Lenvatinib (#19375) and cabozantinib (#18464) were purchased from Cayman Chemical. Trametinib (#S-2673) was purchased from Selleck Chemicals, and buparlisib (#HY-70063) was from MedChem Express. Recombinant human IFNγ (#300-02) was obtained from PeproTech. Cell viability was quantified with Cell Count Reagent SF (nacalai tesque). Absorbance at 450 nm was measured on a micro- plate reader. For quantification of cytotoxicity, we used LDH Cytotoxicity Assay Kit (nacalai tesque). The absorbance value at 490 nm was measured.

### Flow Cytometry

Cells were trypsinized, washed with ice-cold phosphate buffered saline (PBS), and pelleted by centrifugation. Cell pellets were then resuspended in anti-HLA-ABC (clone W6/32, #311403, BioLegend) conjugated to fluorescein isothiocyanate (FITC) (300:1 dilution), anti-β2-microglobulin (B2M) (clone 2M2, #316304, Biolegend) conjugated to FITC (300:1 dilution), or isotype control antibodies. Cells were incubated for 30 min at 4°C. After washing, cells were analyzed using a FACScalibur flow cytometer and CellQuestTM Pro version 6.0 software (both from Becton-Dickinson and Co.).

### Quantitative Real-Time PCR

Total RNA was isolated with Trizol reagent (Invitrogen/Thermo Fisher Scientific) and Direct-zol™ RNA Microprep (Zymo Research). A 500-ng quantity of total RNA was used for the synthesis of first-strand cDNA using a PrimerScript RT cDNA Synthesis Kit (Takara Bio). Individual gene expression was quantified by real-time (RT) PCR using SYBR FAST qPCR Master Mix (KAPA BIOSYSTEMS) and a LightCycler 96 Real-Time PCR system (Roche Diagnostics). Gene expression was normalized to the amount of glyceraldehyde-3-phosphate dehydrogenase (GAPDH) mRNA as an internal control. The primers used for RT-PCR analyses are listed in the **Supplementary Table 1**. The primers were obtained from Invitrogen/Thermo Fisher Scientific.

### Immunoblot Analysis

Harvested HCC cells were homogenized in RIPA buffer, and cellular proteins were then fractionated *via* SDS-PAGE and transferred onto a polyvinylidene fluoride membrane. The membrane was incubated with antibodies against STAT1 (#14994) (1000:1 dilution), phosphorylated STAT1 (Tyr701, #7649) (1000:1 dilution), STAT3 (#9132) (1000:1 dilution), phosphorylated STAT3 (Tyr705, #9131) (250:1 dilution), extracellular signal–regulated kinase (ERK) (#4695) (1000:1 dilution), phosphorylated ERK (Thr202/Tyr204, #9101) (1000:1 dilution), AKT (#9272) (1000:1 dilution), phosphorylated AKT (Ser473, #4060) (250:1 dilution), src homology-containing protein 2 (SHP2) (#3397) (250:1 dilution), phosphorylated SHP2 (Tyr580, #3703) (100:1 dilution) from Cell Signaling Technology; with that against β-actin (#A1978) (1000:1 dilution) from Sigma-Aldrich.

### Immunofluorescent Staining Assay

Cells were incubated on a glass chamber slide with the indicated drug, covered, and incubated with ice-cold 1:1 methanol and acetone mixture for 10 min at −20°C. After washing with PBS, cells were blocked with diluted donkey serum for 30 min at room temperature, then incubated with mouse-anti HLA-ABC antibody (#ab70328, Abcam) and rabbit-anti phosphorylated STAT1 (Tyr701, #7649, Cell Signaling Technology) overnight at 4°C. Cells were washed with PBS and incubated with Alexa Fluor 594-conjugated donkey anti-mouse IgG (#715-585-150, Jackson ImmunoResearch) and 488-conjugated donkey anti-rabbit IgG (#11-545-152, Jackson ImmunoResearch) antibodies, respectively, for 1 hour at room temperature. After washing, slides were mounted with medium containing DAPI (Vectashield H-1500, Vector Laboratories). A BZ-X800 fluorescence microscope (Keyence Corporation) was used to assess the expression and subcellular localization of HLA-ABC and phosphorylated STAT1.

### siRNA Knockdown

Cells were transfected with STAT1 siRNA (#4390824, Thermo Fisher Scientific) or nontargeting siRNA control (#4390843, Thermo Fisher Scientific), using lipofectamine-RNAi max (Life Technologies) and Opti-MEM I (Life Technologies). Reverse transfection was performed according to the manufacturer’s instruction manual. Cells were treated with the indicated drugs, 48 hours after siRNA knockdown, for 72 hours before assaying for surface HLA-I by flow cytometry.

### TCGA Dataset Analysis

Data for HCC patients from The Cancer Genome Analysis (TCGA) were obtained from cBioPortal. Gene expression correlations were assessed according to the Spearman coefficient. Genes associated with T cell inflammation were selected in accordance with a previous report (27).

### Statistical Analysis

Data are expressed as a mean ± SD of results obtained in 3 independent experiments. A two-tailed t test was used to calculate whether observed differences were statistically significant, defined as p < 0.05 (*p < 0.05, **p < 0.01, ***p < 0.001, ****p < 0.0001).

## Results

### Regorafenib Elevates Cell Surface HLA-I Expression in Liver Cancer Cells in the Presence of IFNγ

**Figure 1:**
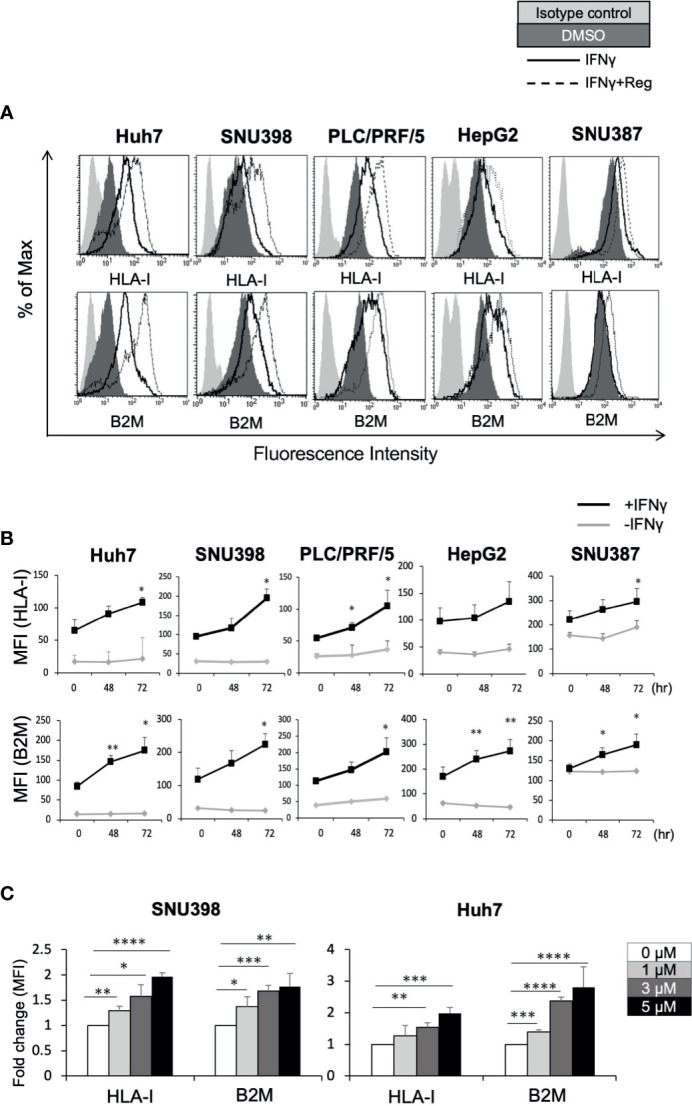
Regorafenib induces expression of cell surface HLA-I molecules in liver cancer cell lines in the presence of IFNγ. **(A)** The indicated cell lines were treated with vehicle (DMSO, dark gray filled), IFNγ (1 ng/mL) alone (black line, unfilled), or IFNγ plus 5 μM regorafenib (black dotted line). Cell surface HLA-I and B2M expression was analyzed by flow cytometry 72 hours later. Cells stained with an isotype control antibody are shown (light gray filled). A representative flow cytometry histogram is shown. **(B)** Cells were treated with regorafenib (5 μM) at the timepoints indicated along the x-axis with or without IFNγ for 72 hours. Values represent the average mean fluorescence intensity (MFI) from 3 independent experiments. **(C)** SNU398 or Huh7 cells were treated with regorafenib at increasing concentrations in the presence of IFNγ for 72 hours. Values are expressed as fold change relative to cells treated with IFNγ alone. Reg, regorafenib. (*: p < 0.05, **: p < 0.01, ***: p < 0.001, ****: p < 0.0001).

The HLA-I molecule is essential for target recognition by CTLs and is a central component of antitumor immunity. B2M is a component of HLA-I and is required for surface presentation and stability of HLA-I on the cell surface. As shown in **Figure 1A**, baseline cell surface HLA-I and B2M expression varied between the 5 liver cancer cell lines, Huh7, SNU398, PLC/PRF/5, HepG2, and SNU387. Since IFNγ is a well-known regulator of HLA-I, we sought to determine whether regorafenib could potentiate the effects of IFNγ on HLA-I expression. We treated liver cancer cell lines with regorafenib in the presence or absence of IFNγ, and evaluated the cell surface levels of HLA-I and B2M protein by flow cytometry. Regorafenib did not affect the basal levels of HLA-I and B2M molecules but had profound effects on IFNγ-induced HLA-I and B2M expression in all of the cell lines examined (**Figure 1B**). This suggests that regorafenib can enhance the upregulation of HLA-I molecules by IFNγ, regardless of the basal level of HLA-I expression. We selected SNU398 and Huh7 cells, which had low basal HLA-I expression, for further evaluation, and treated them with regorafenib at different concentrations in the presence of IFNγ. Flow cytometric analysis revealed that regorafenib influenced surface HLA-I and B2M expression in a dose-dependent manner (**Figure 1C**). We performed cell viability WST-8 assay and cell death/cytotoxicity LDH assay using SNU398 and Huh7 cells. Regorafenib, as well as the other TKIs, decreased cell viability over 72 hours, while additional IFNγ did not affect cell viability (**Supplementary Figure 1A**). The cell death/cytotoxicity assay also showed that regorafenib induced cell death in a dose dependent manner, but additional IFNγ did not affect cell death (**Supplementary Figure 1B**). These results suggest that the effect of dying cells induced by combination treatment on the present findings is not significant, compared with treatment by regorafenib alone.

### Regorafenib Increases HLA-I Gene Expression as Well as the Related Transcriptional Regulators NLRC5 and IRF1

**Figure 2:**
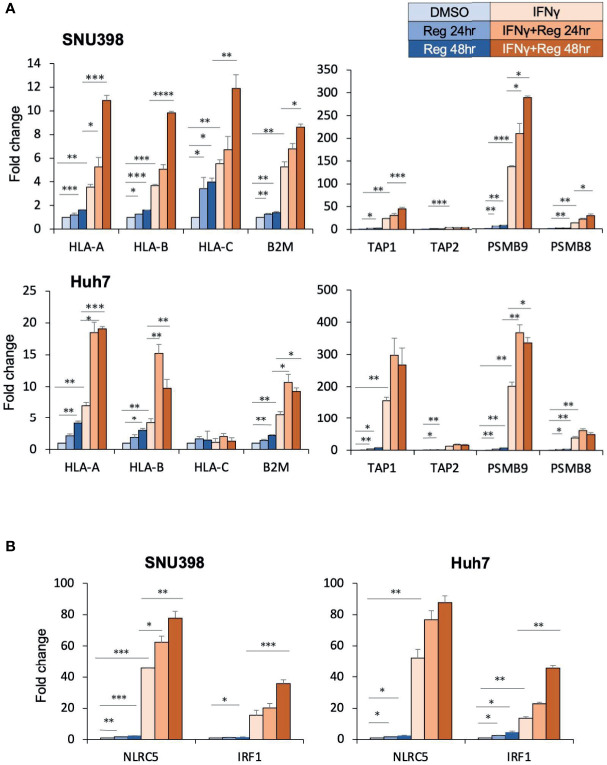
Regorafenib increases gene expression of HLA-I as well as the related transcriptional regulators NLRC5 and IRF1. **(A, B)** SNU398 or Huh7 cells were treated with regorafenib (5 μM) for the indicated times in the absence or presence of IFNγ (1 ng/mL) for 48 hours. mRNA levels were measured using real-time PCR. Data are expressed as fold change relative to vehicle-treated cells. Reg, regorafenib. (*: p < 0.05, **: p < 0.01, ***: p < 0.001, ****: p < 0.0001).

Given that dysregulated cell surface HLA-I expression was reversible, we hypothesized that HLA-I dysregulation might be characterized by transcriptional downregulation rather than genetic alterations (28). There are several critical genes essential for the HLA-I APP, including those encoding immunoproteasome components (proteasomes beta subunits (PSMB) 8 and PSMB9), peptide transporters into the endoplasmic reticulum lumen (transporter associated with antigen processing (TAP)1 and TAP2), HLA-I heavy chains (HLA-ABC), and HLA-I light chain (B2M). These components are substantial to form the HLA-I/peptide complex. Intracellular tumor antigens are processed into peptides by the immunoproteasome, including PSMB. These peptides are subsequently translocated into the endoplasmic reticulum by TAP and bind to the HLA-I complex, which is composed of a heavy chain and β2M. In cancer, one or several proteins in this complex pathway can be dysregulated, resulting in dysfunction of antigen presentation. RT-PCR analysis revealed that regorafenib resulted in significant but smaller increases in the expression of these genes than with IFNγ treatment, and that the combination of IFNγ and regorafenib had greater effects than either alone (**Figure 2A**). Interferon regulatory factor 1 (IRF1) and nucleotide-binding oligomerization domain-like receptor caspase recruitment domain containing protein 5 (NLRC5) are key transcriptional regulators required for the induction of HLA-I APP genes in response to IFNγ stimulation. We found that regorafenib also induced significant increases in both basal and IFNγ-mediated mRNA expression of IRF1 and NLRC5 (**Figure 2B**), suggesting that HLA-I induction by regorafenib might be associated with increased IRF1 and NLRC5.

### Activation of IFNγ/STAT1 Signaling Is Responsible for Regorafenib-Induced HLA-I Expression

**Figure 3:**
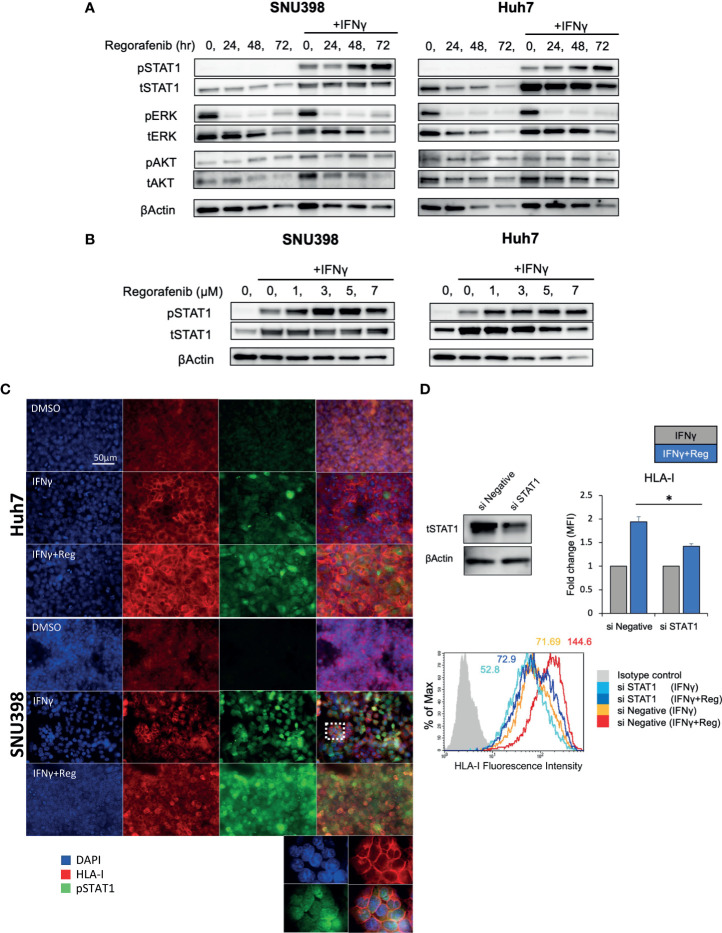
Activation of IFNγ/STAT1 signaling is responsible for regorafenib-induced HLA-I expression. **(A)** SNU398 or Huh7 cells were stimulated by regorafenib (5 μM) at the indicated times with or without IFNγ (1 ng/mL) for 72 hours, and then STAT1, ERK, and AKT protein levels were analyzed by Western blotting. β-actin is used as a loading control. **(B)** Cells were treated with regorafenib at the indicated concentrations over 72 hours with IFNγ. **(C)** Cells were treated with vehicle, IFNγ alone, or IFNγ plus regorafenib for 72 hours. Representative pictures of multiple immunofluorescence staining show the expression of pSTAT1 (green) and HLA-I (red). Scale bar, 50 μm. **(D)** SNU398 cells were transfected with STAT1 siRNA or negative control siRNA over 48 hours, and STAT1 protein levels were analyzed by Western blotting. Then, cells were treated with either IFNγ alone or IFNγ plus regorafenib for 72 hours, and assayed by flow cytometry for surface HLA-I expression. Values are expressed as fold change relative to cells treated with IFNγ alone. A representative flow cytometry histogram is shown. Reg, regorafenib. (*: p < 0.05).

Given that cell surface HLA-I and B2M protein were regulated mainly at the transcriptional level, the upstream signaling pathways regulating HLA-I expression were investigated. Since IFNγ/STAT1 pathway activation has been suggested to have the most potent effect on the expression levels of HLA-I APP genes (28), we next measured the expression of pSTAT1 (Tyr701) and total STAT1 after treatment with regorafenib alone or in addition to IFNγ treatment. As shown in **Figure 3A**, we found that basal levels of pSTAT1 were quite low, despite abundant expression of total STAT1 protein. IFNγ treatment strongly increased expression of both pSTAT1 and total STAT1. Interestingly, after 48-hour treatment with regorafenib, the ability of IFNγ to induce pSTAT1 was significantly augmented. We also observed that IFNγ-induced STAT1 activation was augmented by regorafenib in a dose-dependent manner (**Figure 3B**). A similar observation was confirmed with immunofluorescence staining. As shown in **Figure 3C**, pSTAT1 and HLA-I were greatly upregulated in the cytoplasm and nucleus, and on the cell surface, respectively, after treatment with IFNγ, and further enhanced by the combination of regorafenib and IFNγ. To confirm that HLA-I upregulation induced by regorafenib depends on STAT1 activation, we evaluated cell surface HLA-I expression following STAT1 inhibition by siRNA. Knockdown of STAT1 was performed on SNU398 cells, in addition to treatment with IFNγ alone or IFNγ plus regorafenib. As shown in **Figure 3D**, STAT1 knockdown inhibited upregulation of surface HLA-I expression after regorafenib treatment, suggesting a role for STAT1 in the response to regorafenib. We also evaluated the expression of SHP2, which dephosphorylates pSTAT1 and plays an important role in HLA-associated immune escape in other types of cancers (26), and found that regorafenib had no inhibitory effects on SHP2 gene and protein expression (**Supplementary Figures 2A, B**). STAT3 activation, which has been reported to have opposite effects of STAT1 in terms of antitumor immune response and contribute to a balanced immune response (28) was also tested by immunoblot analysis, but regorafenib did not affect STAT3 activation (**Supplementary Figure 2A**).

### Role of the MEK-ERK Pathway in Induction of HLA-I Expression by Regorafenib

**Figure 4:**
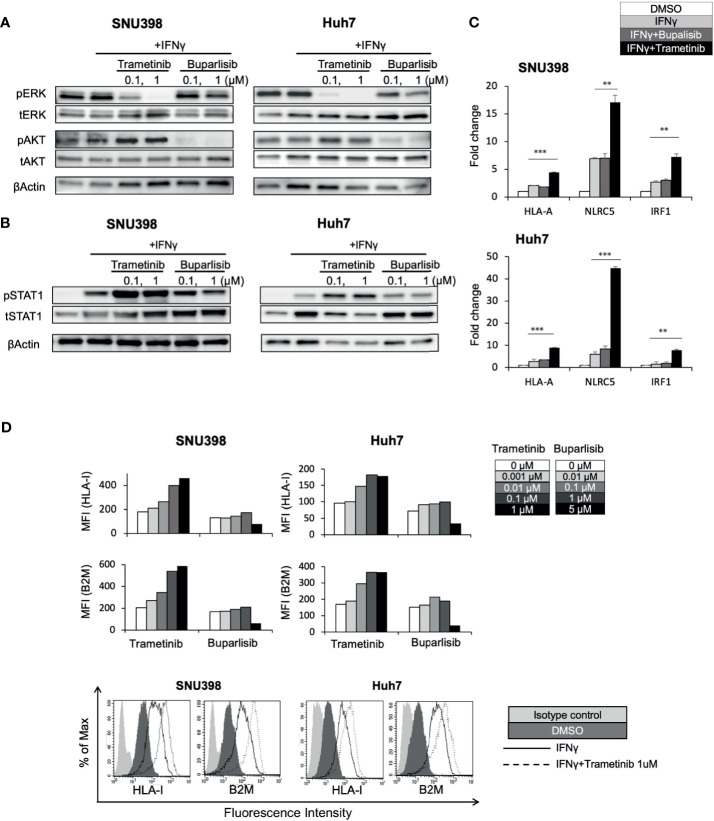
Downregulation of the MEK/ERK pathway is involved in regorafenib-induced STAT1 phosphorylation and HLA-I expression. SNU398 or Huh7 cells were cultured with trametinib or buparlisib at the indicated concentrations in the presence of IFNγ (1 ng/mL) for 24 hours **(A)** or for 72 hours **(B)** and then, ERK, AKT, and STAT1 protein levels were analyzed by Western blotting. β-actin is used as a loading control. **(C)** Cells were treated with buparlisib (0.1 μM) or trametinib (0.1 μM) for 48 hours with IFNγ. HLA-A, NLRC5 and IRF1 mRNA expression was analyzed using real-time PCR. Data are expressed as fold change relative to vehicle-treated cells. **(D)** Cells were treated with trametinib or buparlisib at the indicated concentrations with IFNγ for 72 hours. HLA-I and B2M surface protein expression were analyzed by flow cytometry and presented as MFI. A representative flow cytometry histogram is shown. (**: p < 0.01, ***: p < 0.001).

In other types of cancers, the MAPK pathway has been shown to be a negative regulator of HLA-I expression (21, 22, 24–26). Analysis of 372 HCC tissues from TCGA showed a significant inverse correlation between MAPK1 and HLA-I APP genes expression including HLA-A, HLA-B, HLA-C, and B2M (**Supplementary Figure 3**), highlighting a potential regulatory function of the MAPK pathway on HLA-I expression in HCC. Since our immunoblot analysis revealed that regorafenib strongly inhibited pERK (**Figure 3A**), we hypothesized that downregulation of the MAPK pathway might be associated with regorafenib-induced HLA-I upregulation. The phosphatidylinositol 3-kinase (PI3K)/AKT/mammalian target of rapamycin and the Raf/MEK/ERK signaling are the major mediators of several receptor tyrosine kinase pathways involved in HCC progression. We therefore examined the effects of trametinib, which selectively inhibits the ERK kinase (MEK), on HLA-I expression in HCC cells, compared with the pan-PI3K inhibitor buparlisib. We first confirmed that trametinib and buparlisib inhibited the activation of ERK and AKT, respectively (**Figure 4A**). Immunoblot analysis showed that trametinib markedly augmented STAT1 activation in respond to IFNγ, while buparlisib did not have such effects (**Figure 4B**). We next measured gene expression of HLA-A, NLRC5, and IRF1 after treatment with trametinib and bupalisib in the presence of IFNγ. As shown in **Figure 4C**, expression of these genes was significantly elevated by trametinib, but not by buparlisib. The induction of cell surface HLA-I and B2M expression by IFNγ was enhanced with trametinib in a dose-dependent manner, but not with buparlisib (**Figure 4D**). These results suggest that the inhibition of MAPK signaling augments the IFNγ response pathway and subsequent transcriptional induction of HLA-I molecules in HCC cells. Taken together, downregulation of MAPK signaling might be one of the mechanisms by which regorafenib promotes HLA-I induction.

### Tyrosine Kinase Inhibitors Induce HLA-I Expression *via* the Activated IFNγ/STAT1 Pathway

**Figure 5:**
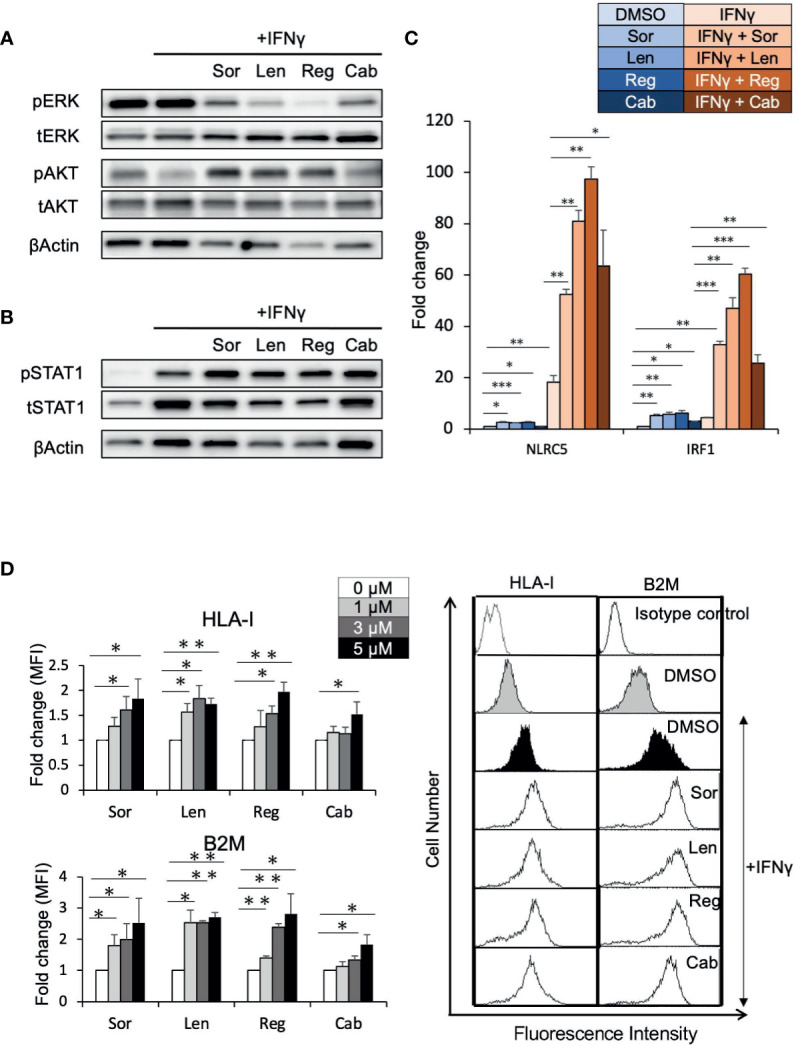
Tyrosine kinase inhibitors induce HLA-I expression by augmenting the IFNγ/STAT1 signaling pathway. **(A, B)** Huh7 cells were treated with each drug at 5 μM in the presence of IFNγ (1 ng/mL) for 24 hours **(A)** or for 72 hours **(B)**. Immunoblotting analysis was performed to detect the protein levels of ERK, AKT, and STAT1. β-actin was used as a loading control. **(C)** Huh7 cells were treated with indicated inhibitors at 5 μM with or without IFNγ over 48 hours. NLRC5 and IRF1 mRNA expression was analyzed using real-time PCR. Data are expressed as fold change relative to vehicle-treated cells. **(D)** Huh7 cells were treated with each drug at the indicated concentrations with IFNγ. Cell surface HLA-I and B2M levels were measured 72 hours later by flow cytometry. The y-axis represents average MFI for 3 independent experiments, expressed as fold change relative to cells treated with IFNγ alone. A representative flow cytometry histogram is shown. Sor, sorafenib; Len, lenvatinib; Reg, regorafenib; Cab, cabozantinib. (*: p < 0.05, **: p < 0.01, ***: p < 0.001).

To determine whether elevated HLA-I expression mediated *via* enhancement of the IFNγ response pathway by regorafenib is a general phenomenon induced by other TKIs, Huh7 cells were treated with sorafenib, lenvatinib, or cabozantinib, as well as regorafenib, in the presence of IFNγ. First, similar suppression of pERK was observed with all of these treatments, while regorafenib showed the strongest inhibition of pERK (**Figure 5A**). We then found that STAT1 activation and subsequent expression of IFNγ response genes were also augmented by all of the TKIs (**Figures 5B, C**). Furthermore, cell surface expression of HLA-I and B2M was found to be significantly enhanced by these TKIs (**Figure 5D**). These results suggest that HLA-I expression in HCC cells may be commonly induced by many different types of TKIs, which inhibit the MAPK pathway.

## Discussion

Several studies have indicated that the approved TKIs for HCC, such as sorafenib, lenvatinib, regorafenib, and cabozantinib, can alter the immune landscape in the tumor microenvironment, indicating the potential for synergy with cancer immunotherapies. In this study, we describe a novel mechanism of TKIs to alter tumor cell phenotype through modification of cell surface molecule expression in HCC cell lines. Our findings reveal that TKIs upregulate cell surface HLA-I and B2M expression *via* augmentation of the IFNγ/STAT1 pathway, and that inhibition of the MAPK pathway by TKIs might be involved in the STAT1 activation. To the best of our knowledge, this is the first study to address the effect of TKIs on HLA-I expression in HCC cells. Potentially, it may be one of the mechanisms by which TKIs enhance the response to ICIs against HCC.

HLA- I expression has a key role in the tumor cell recognition by CTLs. Our flow cytometric analysis showed that the levels of cell surface HLA-I and B2M were greatly induced by regorafenib in the presence of IFNγ. Although we could not confirm whether recognition by CTLs is enhanced by the upregulation of HLA-I and B2M, it has been well established that deficient expression of HLA-I components, including B2M, is a key factor contributing to tumor progression and immunotherapy resistance. Conversely, augmenting the IFNγ response pathway and the expression of HLA-I molecule enhances CTL-mediated anti-tumor immunity in many human cancers (11–13, 29). On the other hand, upregulation of HLA-I molecules on tumor cells is involved in escape from NK cell recognition and, thus, promotes disease progression in tumors that are immunologically controlled mainly by NK cells (30). In HCC, it has been reported that higher HLA-I expression leads to high sensitivity for cytotoxic T lymphocytes and better prognosis in not only HCC cells (18, 19), but also patients with HCC (14, 15). From the analysis of 372 HCC dataset, the expression levels of genes involved in HLA-APP are strongly correlated with those of T cell inflammation (**Supplementary Figure 4**). In addition, a systematic review and meta-analysis of gastrointestinal cancers including HCC also showed that high HLA- I expression was related to a better prognosis (31). We think increased HLA-I expression can play an important role in the combination with TKIs and immunotherapy at least partly. Further studies are needed to prove clinical significance of HLA-I upregulation by TKIs.

In the absence of additional IFNγ stimulation, induction of cell surface HLA-I protein expression was not observed with regorafenib, while regorafenib alone increased HLA-I APP gene expression in HCC cells. This may in part reflect much lower basal expression of NLRC5, IRF1, and HLA-I APP genes, compared with post-IFNγ stimulation, as observed in **Figure 2**. Similarly, decreased IRF1 gene expression has also been reported in Huh7 cells compared with primary hepatocytes (32), and the expression of IRF1 mRNA in HCC tissues is lower than that in adjacent noncancerous tissue (33). Our results suggest that treatment with regorafenib alone affects HLA-I APP genes but is insufficient to upregulate cell surface HLA-I molecules; thus, additional IFNγ stimulation is required to induce cell surface HLA-I expression by regorafenib. IFNγ is a cytokine that is generally present in the tumor microenvironment, which contains CTLs (34). Even if tumors initially lack CTLs, TKIs including regorafenib have the ability to induce CTL infiltration and function by reducing immunosuppressive cells or by normalizing the HCC vasculature (6, 7), resulting in the secretion of IFNγ. Our findings suggest that the upregulation of HLA-I expression by regorafenib might raise a positive-feedback cycle leading to better recognition by CTLs and increased generation of IFNγ.

In the present study, we revealed that the activation of STAT1 is responsible for regorafenib-mediated HLA-I expression. STAT1 is generally considered to be a tumor suppressor and is known to regulate cell survival, proliferation, and immune responses (35). Current research has indicated that the expression of STAT1 is downregulated in a variety of tumor cells, and low STAT1 expression often indicates a poor prognosis for several types of cancers including HCC (36). Recently, Shigeta et al. reported that STAT1 activation is induced by regorafenib in HCCs both *in vitro* and *in vivo* (7), in agreement with our results. These findings suggest that regorafenib directly targets VEGFRs, Tie2, PDGFR and BRAF but may also have an indirect activating effect on STAT1, an immune-relevant target.

The PI3K/AKT signaling and the MEK/ERK signaling are the two major mediators of several receptor tyrosine kinase targeted by regorafenib. Among them, the MEK/ERK pathway has been shown to negatively affect HLA-I protein expression in other types of cancers (21–24). Using the selective MEK inhibitor trametinib and PI3K inhibitor buparlisib, we revealed that inhibition of the MEK/ERK pathway, but not of the PI3K/AKT pathway, enhanced STAT1 activation and subsequent expression of cell surface HLA-I and B2M molecules in HCC cell lines. Consistent with our *in vitro* findings, trametinib has been reported to activate STAT1 and subsequent HLA-I induction in many other types of cancers (24, 37, 38). Notably, ERK has been identified as a transcriptional repressor of IFNγ response genes including STAT1 (39). It has also been shown that ERK is a negative regulator of IFNγ/STAT1 signaling by promoting STAT1 ubiquitination (40). These results suggest that the inhibition of ERK can upregulate STAT1 protein expression, although the mechanism of STAT1 phosphorylation remains unknown. Given that regorafenib directly inhibits Raf/MEK/ERK signaling, it has been suggested that downregulation of this signaling is one of the mechanisms by which regorafenib promotes STAT1 activation. The other TKIs such as sorafenib, lenvatinib, and cabozantinib also showed inhibitory effects on ERK activation. Among these drugs, regorafenib had the most potent effects on HLA-I expression at both the mRNA and cell surface protein levels, possibly dependent on it having the strongest inhibitory activity against ERK. Considering our findings, trametinib, which is already approved for BRAF-mutated melanoma, can greatly potentiate the induction of HLA-I protein expression and, thus, might be a candidate for further clinical study in HCC with the addition of an ICI.

According to previous reports, the upregulation of HLA-I molecules by MAPK pathway inhibition is observed in cells harboring activating mutations in specific MAPK pathway genes. Among the 5 liver cancer cell lines used in this study, 3 cell lines have oncogenic alterations known to activate the RAS/MAPK pathway (Huh7 and SNU387 with FGF19 amplifications; HepG2 with NRAS mutation) (41). However, regardless of these gene alterations, we observed the effects of regorafenib on HLA-I expression in all of the 5 cell lines. This might be due to the constitutive activation of MAPK signaling in HCC being dependent on aberrant upstream signals, inactivation of Raf kinase inhibitor protein, and induction by hepatitis viral proteins (42). This result raises the possibility of regorafenib to benefit a wide variety of HCC cells in terms of the upregulation of HLA-I molecules.

In summary, we have demonstrated that TKIs, those are approved for the treatment of HCC, can augment the IFNγ/STAT1 signaling pathway, and thereby increase the upregulation of cell surface HLA-I molecules in HCC cells. We also found that downregulation of MAPK signaling might be one of the mechanisms by which TKIs promote HLA-I induction. Our findings provide novel evidence that TKIs alter the tumor cell phenotype, which may mediate tumor immune response. Further study is warranted to evaluate the effects of TKIs on HLA-I expression and immune response in HCC animal models and patients. We believe that our findings support the clinical investigation of combination therapy with TKIs and immunotherapies for the treatment of HCC.

## Data Availability Statement

The datasets presented in this study can be found in online repositories. The names of the repository/repositories and accession number(s) can be found in the article/**Supplementary Material**.

## Author Contributions

AT performed experiments, analyzed data and wrote the paper. AT and AU designed the study. AU co-wrote the paper. KoY, SO, SK, KO, YS, KaY, MM, TO, and YI contributed to writing and revision. All authors contributed to the article and approved the submitted version.

## Funding

This research was supported by governmental grants from the Japan Society for the Promotion of Science, Grants-in-Aid for Scientific Research (KAKENHI) #20K08377 (AU), AMED #JP20fk0210059 (AU), #JP19fk0210027 (KY and YI), and #JP19fk0210040 (TO and YI).

## Conflict of Interest

YI received lecture fees from Eisai Co., Ltd., Chugai Pharmaceutical Co., Ltd., Takeda Pharmaceutical Company Limited, and Merck Sharp and Dohme, as well as commercial research funding from Bayer AG, Eisai Co., Ltd., and Merck Sharp and Dohme. MM received lecture fees from Bayer AG, Eisai Co., Ltd, Eli Lilly Japan K.K., Chugai Pharmaceutical Co., Ltd.

The remaining authors declare that the research was conducted in the absence of any commercial or financial relationships that could be construed as a potential conflict of interest.

## Publisher’s Note

All claims expressed in this article are solely those of the authors and do not necessarily represent those of their affiliated organizations, or those of the publisher, the editors and the reviewers. Any product that may be evaluated in this article, or claim that may be made by its manufacturer, is not guaranteed or endorsed by the publisher.

## Supplementary Material

Supplementary Figure 1 Regorafenib induces cell cytotoxicity, regardless of the presence of IFNγ. **(A)** SNU398 or Huh7 cells were treated with each drug at increasing concentrations in the presence or absence of IFNγ (1 ng/mL) for 72 hours and assayed for cell viability by Cell Count Reagenet SF. Data are expressed as fold change relative to vehicle-treated cells. **(B)** Cells were treated with regorafenib at the indicated concentrations with or without IFNγ and assayed for cell cytotoxicity by LDH Cytotoxicity Assay Kit.

Supplementary Figure 2 Regorafenib has no inhibitory activity on SHP2 and STAT3. **(A)** SNU398 or Huh7 cells were stimulated by regorafenib (5 μM) at the indicated times with or without IFNγ (1 ng/mL) for 72 hours, and then SHP2 and STAT3 protein levels were analyzed by Western blotting. Tubulin was used as a loading control. **(B)** Cells were treated with regorafenib at 5 μM with or without IFNγ over 48 hours. SHP2 mRNA expression was analyzed by real-time PCR. Data are expressed as fold change relative to vehicle-treated cells.

Supplementary Figure 3 Gene expression of MAPK1 is negatively correlated with that of HLA-I in TCGA HCC datasets. Correlation between MAPK1 and HLA-A **(A)**, HLA-B **(B)**, HLA-C **(C)**, B2M **(D)** transcripts from 372 HCC samples in TCGA are shown. Data for HCC patients from TCGA were obtained from cBioPortal. Gene expression correlations were assessed according to the Spearman coefficient.

Supplementary Figure 4 Expressions of genes associated with HLA-APP are correlated with those of T cell inflammation in TCGA HCC datasets. Correlation between HLA-APP and T cell inflammation transcripts from 372 HCC samples in TCGA are shown. Data for HCC patients from TCGA were obtained from cBioPortal. Gene expression correlations were assessed according to the Spearman coefficient.

## References

[1] Yang JD Hainaut P Gores JG Amadou A Plymoth A Roberts RL. A Global View of Hepatocellular Carcinoma: Trends, Risk, Prevention and Management. Nat Rev Gastroenterol Hepatol (2019) 16:589–604. 10.1038/s41575-019-0186-y 31439937 PMC6813818

[2] He S Jiang W Fan K Wang X. The Efficacy and Safety of Programmed Death-1 and Programmed Death Ligand 1 Inhibitors for the Treatment of Hepatocellular Carcinoma: A Systematic Review and Meta-Analysis. Front Oncol (2021) 11:626984. 10.3389/fonc.2021.626984 33833987 PMC8021909

[3] Donisi C Puzzoni M Ziranu P Lai E Mariani S Saba G. Immune Checkpoint Inhibitors in the Treatment of HCC. Front Oncol (2020) 10:601240. 10.3389/fonc.2020.601240 33585218 PMC7874239

[4] Finn RS Ikeda M Zhu AX Sung WM Baron DA Kudo M. Phase Ib Study of Lenvatinib Plus Pembrolizumab in Patients With Unresectable Hepatocellular Carcinoma. J Clin Oncol (2020) 38:2960–70. 10.1200/JCO.20.00808 PMC7479760 32716739

[5] Kwilas RA Ardiani A Donahue NR Aftab TD Hodge WJ. Dual Effects of a Targeted Small-Molecule Inhibitor (Cabozantinib) on Immune-Mediated Killing of Tumor Cells and Immune Tumor Microenvironment Permissiveness When Combined With a Cancer Vaccine. J Transl Med (2014) 12:294. 10.1186/s12967-014-0294-y 25388653 PMC4236498

[6] Kato Y Tabata K Kimura T Kinoshita A Ozawa Y Yamada K. Lenvatinib Plus Anti-PD-1 Antibody Combination Treatment Activates CD8+ T Cells Through Reduction of Tumor-Associated Macrophage and Activation of the Interferon Pathway. PloS One (2019) 14:e0212513. 10.1371/journal.pone.0212513 30811474 PMC6392299

[7] Shigeta K Matsui A Kikuchi H Klein S Mamessier E Chen XI. Regorafenib Combined With PD1 Blockade Increases CD8 T-Cell Infiltration by Inducing CXCL10 Expression in Hepatocellular Carcinoma. J Immunother Cancer (2020) 8:e001435. 10.1136/jitc-2020-001435 33234602 PMC7689089

[8] Tsai KA Khan YA Worgo EC Wang LL Liang Y Davila E. A Multikinase and DNA-PK Inhibitor Combination Immunomodulates Melanomas, Suppresses Tumor Progression, and Enhances Immunotherapies. Cancer Immunol Res (2017) 5:790–803. 10.1158/2326-6066.CIR-17-0009 28775208 PMC5626455

[9] Cabrera R Ararat M Xu Y Brusko T Wasserfall C Atkinson AM. Immune Modulation of Effector CD4+ and Regulatory T Cell Function by Sorafenib in Patients With Hepatocellular Carcinoma. Cancer Immunol Immunother (2013) 62:737–46. 10.1007/s00262-012-1380-8 PMC3863727 23223899

[10] Gubin MM Zhang X Schuster H Caron E Ward PJ Noguchi T. Checkpoint Blockade Cancer Immunotherapy Targets Tumour-Specific Mutant Antigens. Nature (2014) 515:577–81. 10.1038/nature13988 PMC4279952 25428507

[11] Zaretsky MJ Garcia-Diaz A Shin SD Escuin-Ordinas H Hugo W Hu-Lieskovan S. Mutations Associated With Acquired Resistance to PD-1 Blockade in Melanoma. N Engl J Med (2016) 375:819–29. 10.1056/NEJMoa1604958 PMC5007206 27433843

[12] Shin SD Zaretsky MJ Escuin-Ordinas H Garcia-Diaz A Hu-Lieskovan S Kalbasi A. Primary Resistance to PD-1 Blockade Mediated by JAK1/2 Mutations. Cancer Discov (2017) 7:188–201. 10.1158/2159-8290.CD-16-1223 27903500 PMC5296316

[13] Gao J Shi ZL Zhao H Chen J Xiong L He Q. Loss of IFN-Gamma Pathway Genes in Tumor Cells as a Mechanism of Resistance to Anti-CTLA-4 Therapy. Cell (2016) 167:397–404. 10.1016/j.cell.2016.08.069 27667683 PMC5088716

[14] Nishibe M Une Y Kobayashi M Kuramitsu Y Hosokawa M Uchino J. HLA Class I Antigens Are Possible Prognostic Factors in Hepatocellular Carcinoma. Int J Oncol (1996) 8:1243–7. 10.3892/ijo.8.6.1243 21544490

[15] Umemoto Y Okano S Matsumoto Y Nakagawara H Matono R Yoshiya S. Prognostic Impact of Programmed Cell Death 1 Ligand 1 Expression in Human Leukocyte Antigen Class I-Positive Hepatocellular Carcinoma After Curative Hepatectomy. J Gastroenterol (2015) 50:65–75. 10.1007/s00535-014-0933-3 24509608

[16] Yoshida S Hazama S Tokuno K Sakamoto K Takashima M Tamesa T. Concomitant Overexpression of Heat-Shock Protein 70 and HLA Class-I in Hepatitis C Virus-Related Hepatocellular Carcinoma. Anticancer Res (2009) 29:539–44. 19331200

[17] Fujiwara K Higashi T Nouso K Nakatsukasa H Kobayashi Y Uemura M. Decreased Expression of B7 Costimulatory Molecules and Major Histocompatibility Complex Class-I in Human Hepatocellular Carcinoma. J Gastroenterol Hepatol (2004) 19:1121–7. 10.1111/j.1440-1746.2004.03467.x 15377288

[18] Akazawa Y Nobuoka D Takahashi M Yoshikawa T Shimomura M Mizuno S. Higher Human Lymphocyte Antigen Class I Expression in Early-Stage Cancer Cells Leads to High Sensitivity for Cytotoxic T Lymphocytes. Cancer Sci (2019) 110:1842–52. 10.1111/cas.14022 PMC6549930 30973665

[19] Matsui M Machida S Itani-Yohda T Akatsuka T. Downregulation of the Proteasome Subunits, Transporter, and Antigen Presentation in Hepatocellular Carcinoma, and Their Restoration by Interferon-Gamma. J Gastroenterol Hepatol (2002) 17:897–907. 10.1046/j.1440-1746.2002.02837.x 12164966

[20] Shen YQ Zhang JQ Xia M Miao FQ Shan XN Xie W. Low-Molecular-Weight Protein (LMP)2/LMP7 Abnormality Underlies the Downregulation of Human Leukocyte Antigen Class I Antigen in a Hepatocellular Carcinoma Cell Line. J Gastroenterol Hepatol (2007) 22:1155–61. 10.1111/j.1440-1746.2006.04421.x 17608862

[21] Watanabe S Hayashi H Haratani K Shimizu S Tanizaki J Sakai K. Et al. Mutational Activation of the Epidermal Growth Factor Receptor Downregulates Major Histocompatibility Complex Class I Expression via the Extracellular Signal-Regulated Kinase in Non–Small Cell Lung Cancer. Cancer Sci (2019) 110:52–60. 10.1111/cas.13860 30390416 PMC6317949

[22] Brea JE Oh YC Manchado E Budhu S Gejman SR Mo G. Kinase Regulation of Human MHC Class I Molecule Expression on Cancer Cells. Cancer Immunol Res (2016) 4:936–47. 10.1158/2326-6066.CIR-16-0177 PMC5110210 27680026

[23] Ebert JP Cheung J Yang Y McNamara E Hong R Moskalenko M. MAP Kinase Inhibition Promotes T Cell and Anti-Tumor Activity in Combination With PD-L1 Checkpoint Blockade. Immunity (2016) 44:609–21. 10.1016/j.immuni.2016.01.024 26944201

[24] Lulli D Carbone LM Pastore S. The MEK Inhibitors Trametinib and Cobimetinib Induce a Type I Interferon Response in Human Keratinocytes. Int J Mol Sci (2017) 18:2227. 10.3390/ijms18102227 PMC5666906 29064427

[25] Sapkota B Hill EC Pollack PB. Vemurafenib Enhances MHC Induction in BRAFV600E Homozygous Melanoma Cells. OncoImmunology (2013) 2:e22890. 10.4161/onci.22890 23483066 PMC3583938

[26] Srivastava MR Trivedi S Concha-Benavente F Hyun-Bae J Wang L Seethala RR. STAT1-Induced HLA Class I Upregulation Enhances Immunogenicity and Clinical Response to Anti-EGFR mAb Cetuximab Therapy in HNC Patients. Cancer Immunol Res (2015) 3:936–45. 10.1158/2326-6066.CIR-15-0053 PMC4526378 25972070

[27] Zhou Y Bastian NI Long DM Dow M Li W Liu T. Activation of NF-kB and P300/CBP Potentiates Cancer Chemoimmunotherapy Through Induction of MHC-I Antigen Presentation. Proc Natl Acad Sci USA (2021) 118:e2025840118. 10.1073/pnas.2025840118 33602823 PMC7923353

[28] Cornel MA Mimpen LI Nierkens S. MHC Class I Downregulation in Cancer: Underlying Mechanisms and Potential Targets for Cancer Immunotherapy. Cancers (2020) 12:1760. 10.3390/cancers12071760 PMC7409324 32630675

[29] Patel JS Sanjana EN Kishton JR Eidizadeh A Vodnala KS Cam M. Identification of Essential Genes for Cancer Immunotherapy. Nature (2017) 548:537–42. 10.1038/nature23477 PMC5870757 28783722

[30] Ljunggren GH Karre K. In Search of the 'Missing Self': MHC Molecules and NK Cell Recognition. Immunol Today (1990) 11:237–44. 10.1016/0167-5699(90)90097-s 2201309

[31] Najafimehr H Hajizadeh N Nazemalhosseini-Mojarad E Pourhoseingholi AM Abdollahpour-Alitappeh M Ashtari S. The Role of Human Leukocyte Antigen Class I on Patient Survival in Gastrointestinal Cancers: A Systematic Review and Meta-Analysis. Sci Rep (2020) 10:728. 10.1038/s41598-020-57582-x 31959894 PMC6970991

[32] Wan PQ Zhang JH Du Q Dong K Luo J Heres C. Analysis of the Relationship Between microRNA-31 and Interferon Regulatory Factor-1 in Hepatocellular Carcinoma Cells. Eur Rev Med Pharmacol Sci (2020) 24:647–54. 10.26355/eurrev_202001_20041 32016965

[33] Moriyama Y Nishiguchi S Tamori A Koh N Yano Y Kubo S. Tumor-Suppressor Effect of Interferon Regulatory Factor-1 in Human Hepatocellular Carcinoma. Clin Cancer Res (2001) 7:1293–8. 11350897

[34] Hu Y Sun H Zhang H Wang X. An Immunogram for an Individualized Assessment of the Antitumor Immune Response in Patients With Hepatocellular Carcinoma. Front Oncol (2020) 10:1189. 10.3389/fonc.2020.01189 32850343 PMC7413104

[35] Meissl K Macho-Maschler S Müller M Strobl B. The Good and the Bad Faces of STAT1 in Solod Tumours. Cytokine (2017) 89:12–20. 10.1016/j.cyto.2015.11.011 26631912

[36] Chen G Wang H Xie S Ma J Wang G. STAT1 Negatively Regulates Hepatocellular Carcinoma Cell Proliferation. Oncol Rep (2013) 29:2303–10. 10.3892/or.2013.2398 23588992

[37] Kang SH Keam B Ahn YO Park HR Kim M Kim MT. Inhibition of MEK With Trametinib Enhances the Efficacy of Anti-PD-L1 Inhibitor by Regulating Anti-Tumor Immunity in Head and Neck Squamous Cell Carcinoma. Oncoimmunology (2019) 8:e1515057. 10.1080/2162402X.2018.1515057 30546955 PMC6287796

[38] Franklin AD James LJ Axelrod LM Balko MJ. MEK Inhibition Activates STAT Signaling to Increase Breast Cancer Immunogenicity via MHC-I Expression. Cancer Drug Resist (2020) 3:603–12. 10.20517/cdr.2019.109 PMC7556720 33062958

[39] Hu S Xie Z Onishi A Yu X Jiang L Lin J. Profiling the Human Protein-DNA Interactome Reveals ERK2 as a Transcriptional Repressor of Interferon Signaling. Cell (2009) 30:610–22. 10.1016/j.cell.2009.08.037 PMC2774939 19879846

[40] Zhang Y Chen Y Liu Z Lai R. ERK Is a Negative Feedback Regulator for IFN-γ/STAT1 Signaling by Promoting STAT1 Ubiquitination. BMC Cancer (2018) 31:613. 10.1186/s12885-018-4539-7 PMC5984314 29855346

[41] Caruso S Calatayud AL Pilet J Bella LT Rekik S Imbeaud S. Analysis of Liver Cancer Cell Lines Identifies Agents With Likely Efficacy Against Hepatocellular Carcinoma and Markers of Response. Gastroenterology (2019) 157:760–76. 10.1053/j.gastro.2019.05.001 31063779

[42] Chen C Wang G. Mechanisms of Hepatocellular Carcinoma and Challenges and Opportunities for Molecular Targeted Therapy. World J Hepatol (2015) 7:1964–70. 10.4254/wjh.v7.i15.1964 PMC4517155 26244070

