# Supplementary figures and images for: Tyrosine Kinase Inhibitors Stimulate HLA Class I Expression by Augmenting the IFNγ/STAT1 Signaling in Hepatocellular Carcinoma Cells
Source: Front Oncol. 2021 Aug 11;11:707473. doi: 10.3389/fonc.2021.707473 (PMC8385668; doi:10.3389/fonc.2021.707473)

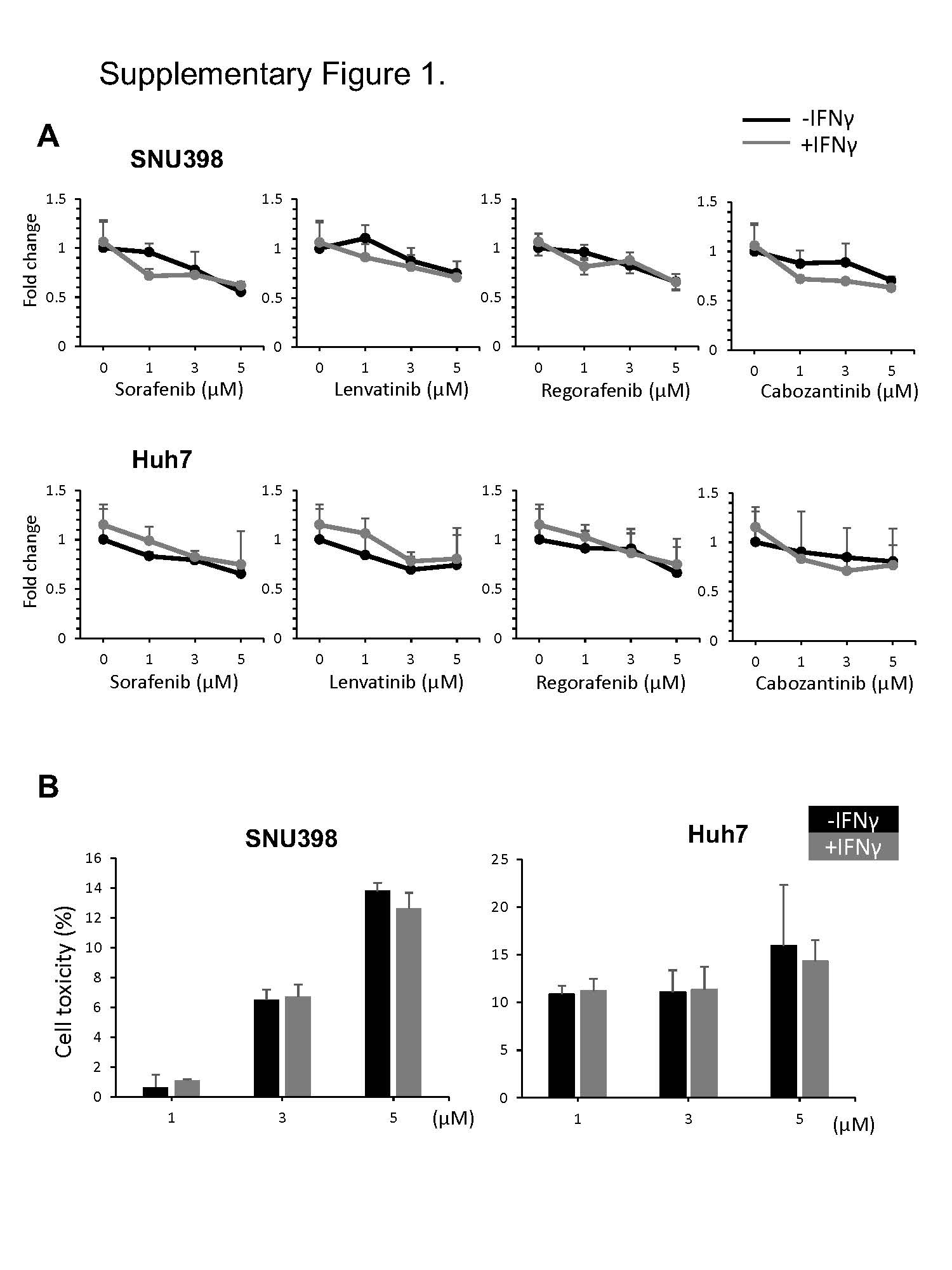

Supplement: Supplementary Figure 1 — Regorafenib induces cell cytotoxicity, regardless of the presence of IFNγ. (A) SNU398 or Huh7 cells were treated with each drug at increasing concentrations in the presence or absence of IFNγ (1 ng/mL) for 72 hours and assayed for cell viability by Cell Count Reagenet SF. Data are expressed as fold change relative to vehicle-treated cells. (B) Cells were treated with regorafenib at the indicated concentrations with or without IFNγ and assayed for cell cytotoxicity by LDH Cytotoxicity Assay Kit. [file Image_1.jpeg]

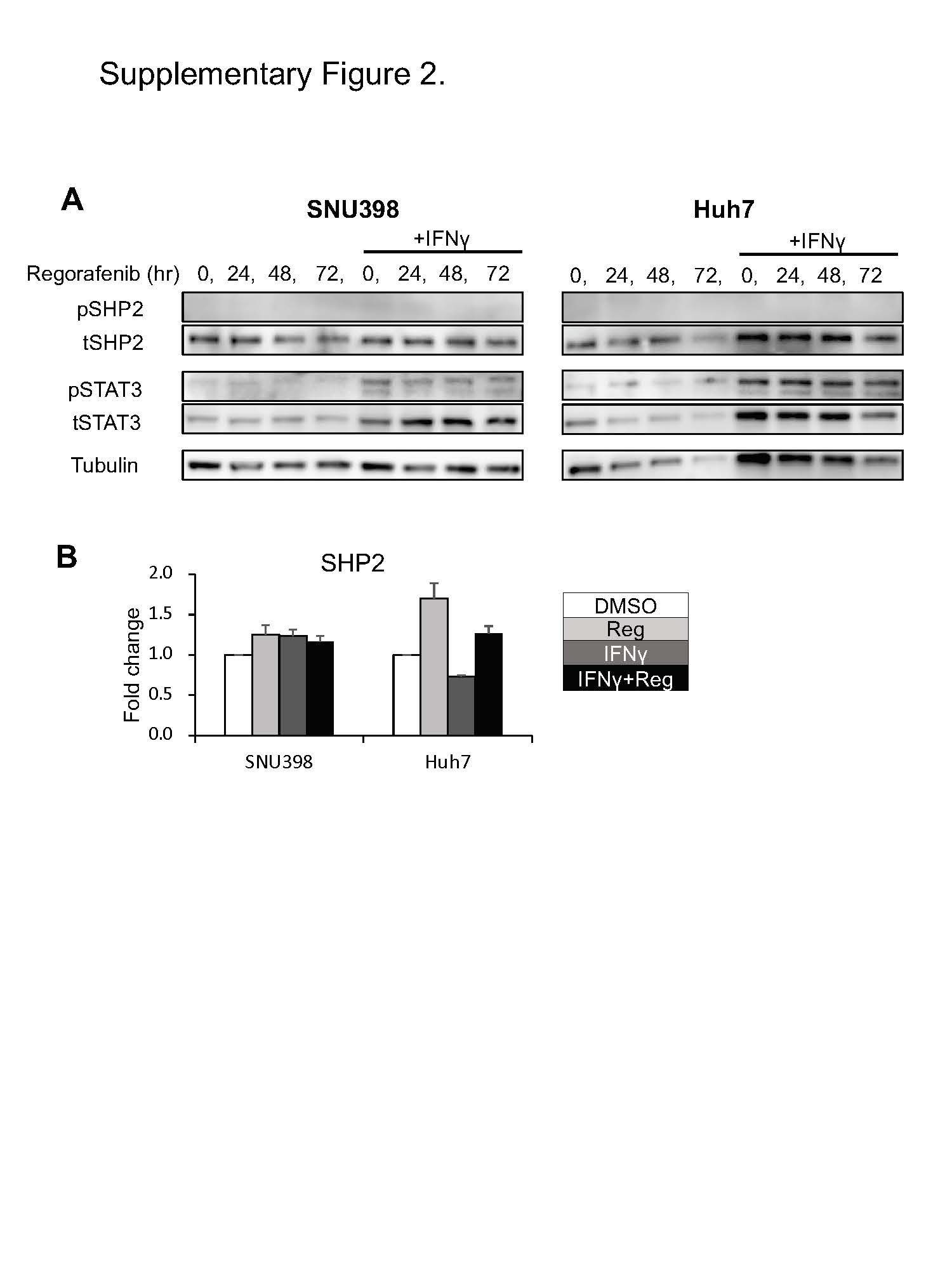

Supplement: Supplementary Figure 2 — Regorafenib has no inhibitory activity on SHP2 and STAT3. (A) SNU398 or Huh7 cells were stimulated by regorafenib (5 μM) at the indicated times with or without IFNγ (1 ng/mL) for 72 hours, and then SHP2 and STAT3 protein levels were analyzed by Western blotting. Tubulin was used as a loading control. (B) Cells were treated with regorafenib at 5 μM with or without IFNγ over 48 hours. SHP2 mRNA expression was analyzed by real-time PCR. Data are expressed as fold change relative to vehicle-treated cells. [file Image_2.jpeg]

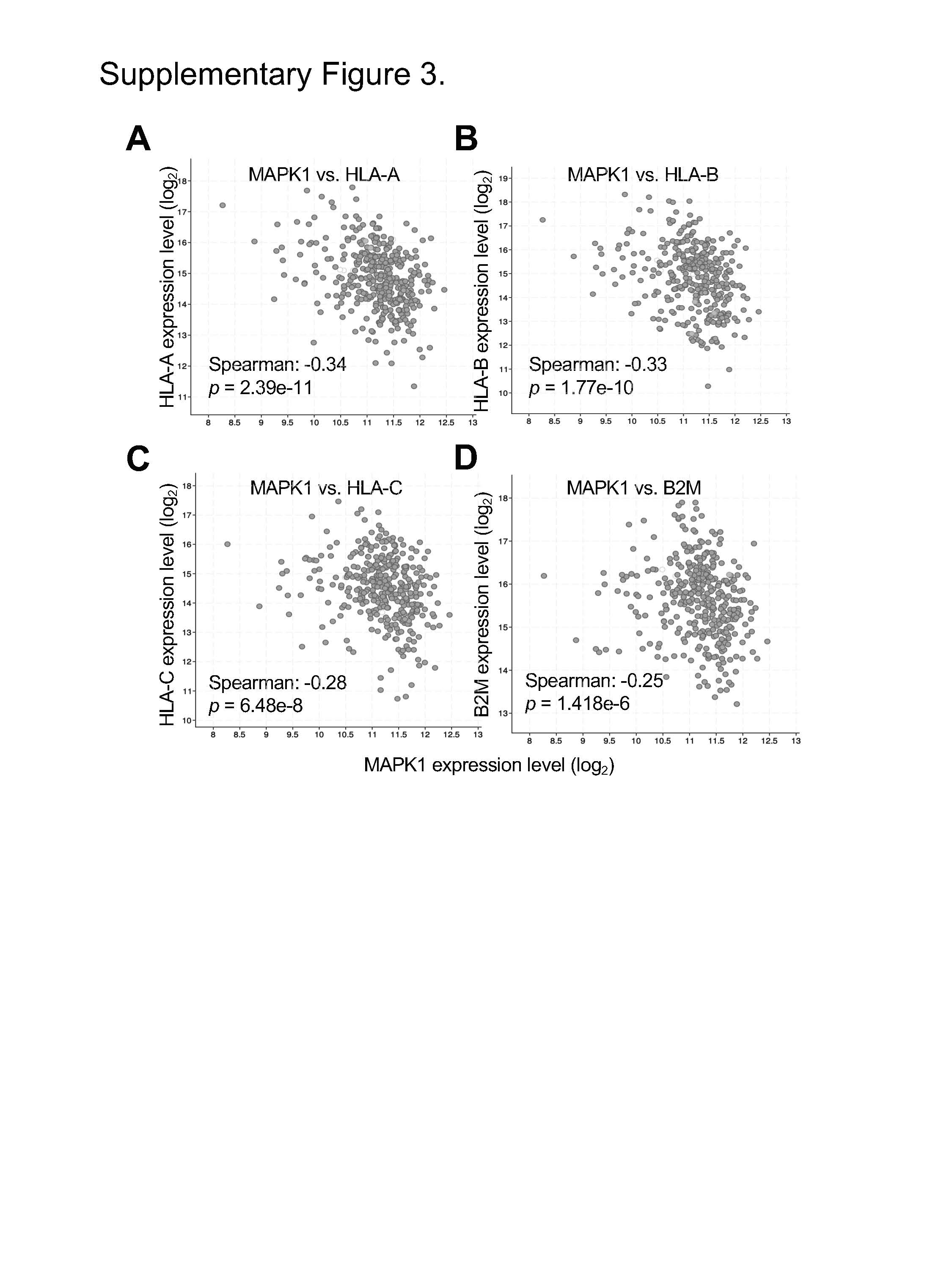

Supplement: Supplementary Figure 3 — Gene expression of MAPK1 is negatively correlated with that of HLA-I in TCGA HCC datasets. Correlation between MAPK1 and HLA-A (A), HLA-B (B), HLA-C (C), B2M (D) transcripts from 372 HCC samples in TCGA are shown. Data for HCC patients from TCGA were obtained from cBioPortal. Gene expression correlations were assessed according to the Spearman coefficient. [file Image_3.jpeg]

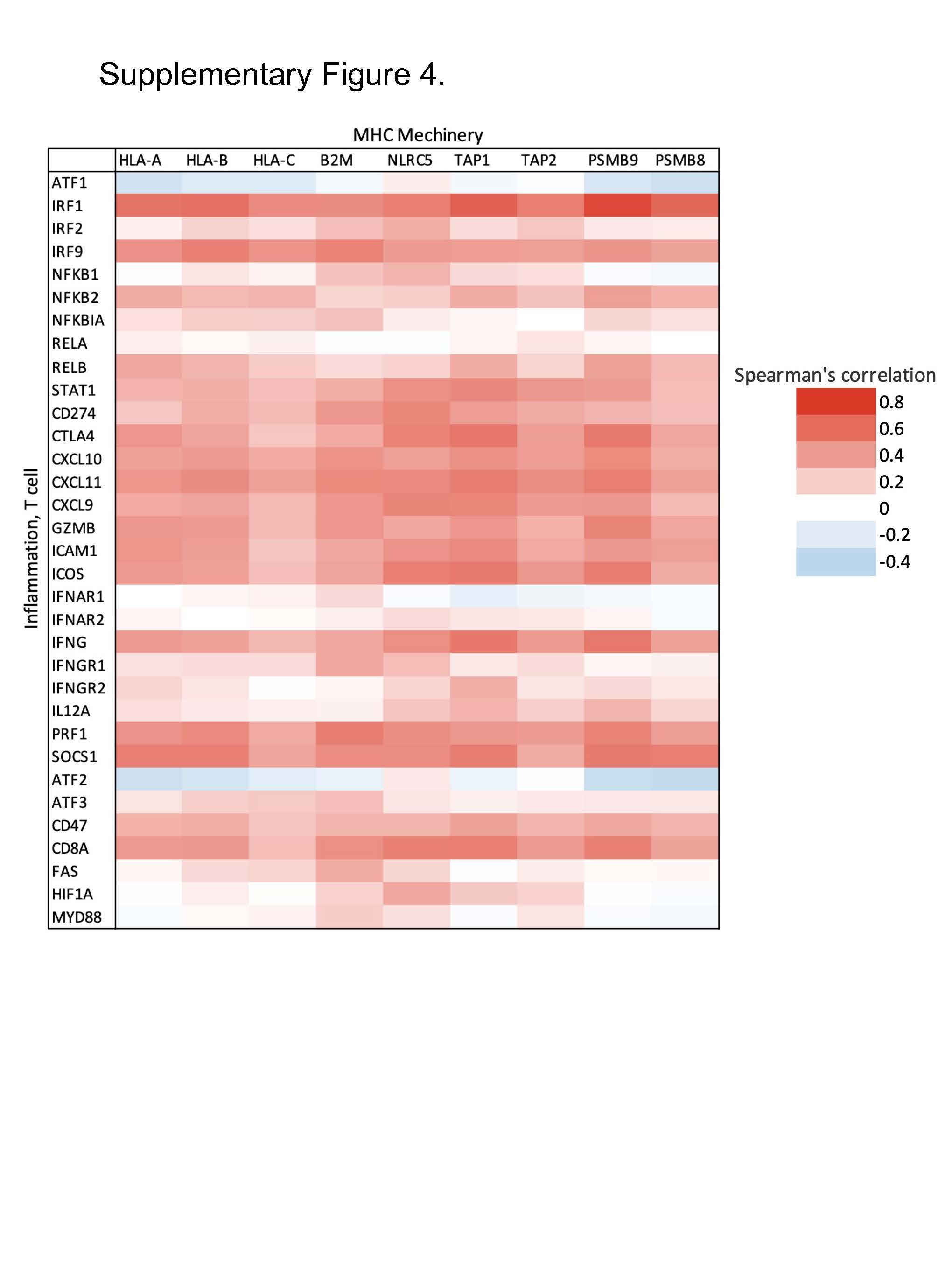

Supplement: Supplementary Figure 4 — Expressions of genes associated with HLA-APP are correlated with those of T cell inflammation in TCGA HCC datasets. Correlation between HLA-APP and T cell inflammation transcripts from 372 HCC samples in TCGA are shown. Data for HCC patients from TCGA were obtained from cBioPortal. Gene expression correlations were assessed according to the Spearman coefficient. [file Image_4.jpeg]
