# Supplementary material for: Tyrosine Kinase Inhibitors Stimulate HLA Class I Expression by Augmenting the IFNγ/STAT1 Signaling in Hepatocellular Carcinoma Cells
Source: Front Oncol. 2021 Aug 11;11:707473. doi: 10.3389/fonc.2021.707473 (PMC8385668; doi:10.3389/fonc.2021.707473)
Supplement: Supplementary file 5 [file Table_1.docx]

# Table S1. List of primers used for quantitative RT-PCR

| **Species** | **Target Gene** | **Sequence (5' - 3')** | |
| --- | --- | --- | --- |
| human | *HLA-A* | forward | AAAAGGAGGGAGTTACACTCAGG |
| human | *HLA-A* | reverse | GCTGTGAGGGACACATCAGAG |
| human | *HLA-B* | forward | CAGTTCGTGAGGTTCGACAG |
| human | *HLA-B* | reverse | CAGCCGTACATGCTCTGGA |
| human | *HLA-C* | forward | CCATGAGGTATTTGTGGACCG |
| human | *HLA-C* | reverse | TCTCGGACTCTCGTCGTCG |
| human | *B2M* | forward | GAGGCTATCCAGCGTACTCCA |
| human | *B2M* | reverse | CGGCAGGCATACTCATCTTTT |
| human | *TAP1* | forward | TGCCCCGCATATTCTCCCT |
| human | *TAP1* | reverse | CACCTGCGTTTTCGCTCTTG |
| human | *TAP2* | forward | TGGACGCGGCTTTACTGTG |
| human | *TAP2* | reverse | GCAGCCCTCTTAGCTTTAGCA |
| human | *PSMB9* | forward | GCACCAACCGGGGACTTAC |
| human | *PSMB9* | reverse | CACTCGGGAATCAGAACCCAT |
| human | *PSMB8* | forward | CACGCTCGCCTTCAAGTTC |
| human | *PSMB8* | reverse | AGGCACTAATGTAGGACCCAG |
| human | *NLRC5* | forward | ACAGCATCCTTAGACACTCCG |
| human | *NLRC5* | reverse | CCTTCCCCAAAAGCACGGT |
| human | *IRF1* | forward | ATGCCCATCACTCGGATGC |
| human | *IRF1* | reverse | CCCTGCTTTGTATCGGCCTG |
| human | *GAPDH* | forward | TGACAACTTTGGTATCGTGGAAGG |
| human | *GAPDH* | reverse | AGGCAGGGATGATGTTCTGGAGAG |
| human | *SHP2* | forward | AAAGGGGAGAGCAATGACGG |
| human | *SHP2* | reverse | CTCCACCAACGTCGTATTTCA |
